# Neonatal Outcomes in Patients with Gestational Diabetes Mellitus Treated with Metformin: A Retrospective Study in Saudi Arabia

**DOI:** 10.3390/biomedicines12092040

**Published:** 2024-09-07

**Authors:** Khaled H. Aburisheh, Mazen M. Barhoush, Abdulaziz N. Alahmari, Ziyad A. Altasan, Muffarah H. Alharthi

**Affiliations:** 1 University Diabetes Center, King Saud University Medical City, King Saud University, Riyadh 11472, Saudi Arabia; mazen_barhoush@yahoo.com; 2 Department of Family Medicine, King Saud University Medical City, Riyadh 11472, Saudi Arabia; abdulaziznh94@gmail.com; 3 Ministry of Health, Riyadh 11472, Saudi Arabia; ziyadtasan@gmail.com; 4 Department of Family and Community Medicine, College of Medicine, University of Bisha, Bisha 61922, Saudi Arabia; muffarah@hotmail.com

**Keywords:** gestational diabetes, neonatal outcomes, metformin, insulin, diet

## Abstract

Background: Gestational diabetes mellitus (GDM) is a common endocrine disease that can occur during pregnancy, increasing the risk of fetal morbidity and mortality. Metformin is a commonly used therapeutic approach for managing GDM. However, there is controversy regarding the effects of metformin on fetal outcomes during pregnancy. This study aimed to evaluate the safety of metformin in relation to neonatal complications, compared to treatment with insulin and/or specialized diets. Method: This was a retrospective study that included pregnant women who were diagnosed with GDM and treated with specialized diets, metformin, or insulin. Data were collected from patients’ electronic medical records and analyzed to evaluate the risk of neonatal outcomes in the metformin group compared to the others. Results: The study included 234 women with GDM. There was no difference between the metformin and insulin groups in terms of the rates of neonatal outcomes, while neonatal hypoglycemia, neonatal hyperbilirubinemia, large for gestational age, and respiratory distress were higher in the metformin group when compared to the diet group. Metformin slightly increased the risk of a lower APGAR score compared to diet alone. Conclusions: Metformin was found to be a safe therapy for the fetus when used to manage GDM, compared to insulin therapy. More randomized studies are needed to confirm these findings in the Saudi population.

## 1. Introduction

Gestational diabetes mellitus (GDM) to refers to any abnormally high glucose value that is diagnosed during pregnancy [1]; however, while this term is typically reserved for women diagnosed in the second or third trimester, it can be diagnosed before that period [2]. The development of GDM is considered the most frequent endocrine disorder in pregnancy, and it is usually associated with adverse outcomes that affect both the mother and the fetus [3]. The overall prevalence of GDM in the Middle East and North Africa has been found to be 13%, and is slightly higher in Arab Gulf countries (14.7%) [4].

Women with GDM have higher incidence of hypertension (HTN), pre-eclampsia, preterm birth, and cesarian section, as well as increased risk of the development of type 2 diabetes mellitus (T2DM) after delivery. Moreover, there is an increased risk of fetal outcomes including higher birth weight, macrosomia, polyhydramnios, birth injury, neonatal hypoglycemia, and neonatal hyperbilirubinemia [5,6].

Early intervention aimed at achieving recommended blood glucose levels may help to delay short-term complications. The primary strategy for managing GDM involves promoting a nutritious diet and advocating for regular physical activity, which could be effective in achieving normal blood glucose levels for approximately 70% of pregnant individuals with GDM [7]. However, in those with uncontrolled levels, the next step is to consider the addition of pharmacological therapy as a means to control hyperglycemia and prevent both maternal and infant complications [8].

Insulin has traditionally been regarded as the standard treatment. This recommendation is consistently supported by the American Diabetes Association (ADA) guidelines [8]. However, it necessitates multiple daily injections and requires patients to undergo training in the technical aspects of treatment. This can result in increased weight gain and higher medical costs. Additionally, approximately 70% of women who use insulin during pregnancy experience episodes of hypoglycemia [9].

Metformin primarily targets the mitochondria, to decrease hepatic gluconeogenesis, regulate hepatic function influenced by glucagon, and improve peripheral insulin sensitivity [10]. Although there is a lack of comprehensive data on the long-term effects of metformin use during pregnancy on offspring as it can cross the placenta [11], it is frequently chosen as the initial treatment option for GDM patients with mild hyperglycemia. Metformin is more cost-effective compared to insulin, has a lower risk of hypoglycemia, and can help to reduce maternal gestational weight gain as well as the occurrence of infants born large for gestational age (LGA) [12]. The use of metformin in pregnancy remains a topic of debate and controversy. While certain studies support its use for glycemic control in pregnant women, others have indicated an elevated risk of adverse events [13,14]. Moreover, to the best of our knowledge, there is no such study considering the Saudi pregnant population diagnosed with GDM.

The present study aimed to assess the neonatal outcomes in Saudi GDM patients treated with metformin compared to diet alone or insulin therapy.

## 2. Materials and Methods

### 2.1. Ethical Approval

This study was conducted according to the relevant guidelines and regulations, and the Institutional Review Board of the College of Medicine, King Saud University, Saudi Arabia reviewed and approved the protocol (E-22-6911). As this was a retrospective data collection study, informed consent was waived by the Institutional Review Board of the College of Medicine, King Saud University, Riyadh.

### 2.2. Study Design

A retrospective observational study was conducted through a review of the medical records of women with GDM who attended gestational diabetes clinics and delivered their child at King Saud University Medical City (KSUMC) in Riyadh, Saudi Arabia, from January 2017 to June 2022.

### 2.3. Diagnosis and Management of GDM

GDM was diagnosed using a 75 g oral glucose tolerance test (OGTT), with one diagnostic criterion used throughout the study period—either a fasting plasma glucose level of ≥5.1 mmol/L, a 1-h plasma glucose level of ≥10 mmol/L, or a 2-h plasma glucose level of ≥8.5 mmol/L—according to ADA guidelines [15]. After the diagnosis of GDM, patients were referred to diabetes educators and dietitians for dietary advice, including information about a carbohydrate-modified diet. Patients were also advised to monitor their blood glucose levels at least 4 times daily (when fasting and at 2-h post-meals) using a blood glucose meter. Patients were evaluated by an endocrinologist or diabetologist, and management was modified according to the target blood glucose levels (i.e., fasting < 5.3 mmol/L and 2 h post-prandial < 6.7 mmol/L) [8]. Pharmacological therapy was started if the target blood glucose level was not achieved through diet and lifestyle modification alone. The choice of pharmacological therapy between metformin and insulin was dependent on the physician’s recommendation and the patient’s preference. If metformin was started but was not sufficient to ameliorate hyperglycemia, insulin therapy was initiated.

### 2.4. Subjects

GDM patients who attended gestational diabetes clinics, delivered their child at KSUMC in Riyadh, and had a complete medical record were collected randomly from the full list covering the study period, consisting of 1607 GDM patients, and were included in the study. We excluded patients with type 1 or type 2 diabetes mellitus, patients with any other disease that could affect blood glucose control (e.g., autoimmune and inflammatory diseases), and those using corticosteroids. Moreover, we excluded those who were treated with a combination of metformin and insulin.

The minimum sample size needed for this study was estimated to be 234 patients based on α = 0.05, β = 0.99, an odds ratio (OR) of 1 to assess neonatal outcomes, and a binomial distribution. The patients were divided into 3 groups according to the means of GDM management (78 patients in the diet-only group, 78 in the insulin-only group, and 78 in the metformin-only group).

### 2.5. Data Collection

Data were extracted from the patients’ electronic medical records, including maternal demographics (i.e., age at pregnancy; body mass index (BMI) in the first trimester; family history ofT2DM; parity; previous history of GDM; previous history of abortions; previous history of macrosomia; gestational age at diagnosis of GDM; gestational age at initiation of pharmacotherapy; OGTT results; glycated hemoglobin (HbA1c) before delivery; the averages of fasting blood sugar (FBS) and 2-h post-prandial glucose (PPG), both calculated from documented home glucose readings before delivery; pre-pregnancy and pregnancy-induced HTN; pre-eclampsia; and gestational age at delivery) and neonatal outcomes (i.e., preterm birth (<37 weeks); neonatal intensive care unit (NICU) admission; neonatal hypoglycemia (<45 mg/dL) and hypocalcemia (<9 mg/dL); congenital anomalies; birth injury; birth weight; perinatal death; five-minute APGAR score at 5 min—this method helps to identify infants requiring respiratory support or other resuscitative measures (with a score of 7–10 considered as reassuring, a score of 4–6 as moderately abnormal, and a score of 0–3 as low); macrosomia, defined as an instance where a fetus is larger than 4000 g; shoulder dystocia; neonatal jaundice or hyperbilirubinemia, where total serum bilirubin is more than 12 mg/dL or is treated with phototherapy; respiratory distress; LGA, defined as birth weight >90th centile for gestational age and gender; and small for gestational age (SGA), defined as birth weight <10th centile for gestational age and gender).

### 2.6. Statistical Analysis

The analysis was performed using PSPP software (version 1.6.2-g78a33a). Data are presented as the mean and SD or the number and percentage for numerical and categorical variables, respectively. Comparisons were made between all three groups (overall test), and pair-wise comparisons were made between mothers treated with metformin and insulin, as well as between mothers treated with metformin and diet. Differences between groups were compared using Student’s independent *t*-test and one-way analysis of variance (ANOVA) for continuous data, and the chi-squared test for categorical data. To evaluate the independent effect of metformin on neonatal outcomes, multivariate logistic regression was performed to estimate adjusted ORs and CIs. We considered *p*-values lower than 0.05 to be statistically significant.

## 3. Results

### 3.1. Study Population and Demographics

During the study period of January 2017 to June 2022, 234 women with GDM were included in the study. They were categorized equally into three groups, according to their management regimen (i.e., metformin, insulin, or diet). The mean age of all participants was 33.09 ± 5.57 years. Patients who were using metformin were significantly older than those who were treated through diet alone. Most of the patients were obese during the first trimester, with a mean BMI of 33.34 ± 5.47 kg/m^2^. There was no significant difference between the metformin and insulin groups; however, the metformin group was significantly heavier than the diet group. Moreover, metformin users were diagnosed with GDM earlier than those who were treated through diet alone, with the mean gestational age of GDM diagnosis for all patients being 27.16 ± 4.51 weeks. The presence of a family history of T2DM, previous macrosomia, and history of abortions was similar in all groups. However, women treated with metformin had more history of previous GDM than those treated through diet, and less history than those treated with insulin. None of the participants had pre-pregnancy HTN, and only a few metformin- or insulin-treated patients developed HTN and pre-eclampsia during pregnancy (3% and 2.1%, respectively), while no patients in the diet group developed these conditions. Furthermore, the mean gestational age of delivery was 38 ± 1.67 weeks and was significantly earlier in the metformin group compared to the diet group (Table 1).

### 3.2. Glycemic Control

Table 1 presents the OGTT values at diagnosis of GDM, where the mean of fasting and the 1-h and 2-h post-OGTT glucose levels were 5.34 ± 1.19, 10.87 ± 1.85, and 9.16 ± 2.11 mmol/L, respectively. The highest FBS values were observed among the insulin group while the lowest were in the diet group, with significant differences when compared to the metformin group. Furthermore, the metformin group had significantly lower 1-h post-OGTT glucose levels compared to the insulin group (10.73 ± 1.95 vs. 11.39 ± 2.07 mmol/L; *p* = 0.043) and higher 2-h post-OGTT glucose levels compared to the diet group (9.42 ± 2.13 vs. 8.67 ± 2.07 mmol/L; *p* = 0.027).

There was no significant difference in HbA1c between the groups before delivery, with a mean HbA1c of 6.41 ± 3.7%; however, the averages of FBS and 2-h PPG were significantly higher in the metformin and insulin groups compared to the diet group.

### 3.3. Neonatal Outcomes

Table 2 presents the neonatal outcomes for all therapeutic groups. There were no significant differences between the insulin and metformin groups for all neonatal outcome parameters analyzed in this study. Women treated with metformin had a significantly higher rate of neonatal hypoglycemia compared to those treated with diet alone (9 vs. 1.3%; *p* = 0.029). Moreover, neonatal jaundice and hyperbilirubinemia, respiratory distress, and LGA were significantly higher in the metformin group compared to the diet group (26.9 vs. 14.1%, *p* = 0.047; 9 vs. 1.3%, *p* = 0.029; 9 vs. 1.3%, *p* = 0.029, respectively).

### 3.4. Multiple Regression Analysis

After multiple regression analyses with the adjustment of multiple factors, metformin did not increase the risk of neonatal outcomes when compared to insulin and diet, except for the five-minute APGAR score, where metformin significantly increased the risk of a lower score compared to diet (standardized beta = −0.18; *p* = 0.036) (Table 3).

## 4. Discussion

Our study revealed that there is no significant difference between the effects of metformin and insulin, as therapies for GDM, on neonatal outcomes. Moreover, we found significantly higher rates of neonatal hypoglycemia, neonatal hyperbilirubinemia, respiratory distress, and LGA in the metformin-treated group compared to the diet-only treatment group. However, from the multiple regression analysis, we concluded that metformin has no significant effect on the neonatal outcome, aside from a slightly increased risk of a lower APGAR score when compared to diet alone.

The results of our study indicate that women who were started on metformin were heavier, older, and had earlier diagnoses of GDM than those who received treatment through diet alone. Moreover, OGTT values, average FBS, and PPGlevels were higher in the metformin group. These findings are not surprising, as in people who are obese higher measures of non-esterified fatty acids, glycerol, counterregulatory hormones, and pro-inflammatory cytokines that partake in the advancement of insulin resistance are delivered by adipose tissue [16]. This leads to higher glucose values, and reflects the routine clinical practice of choosing metformin in such patients. Similar data have been reported in many other studies [17,18,19,20,21]. 

Our study revealed that the metformin-treated group had a higher prevalence of previous GDM when compared to the diet control group, but a lower prevalence compared to the insulin-treated group. This suggests that the need for escalated medication may be associated with insulin resistance. Moreover, the history of previous GDM is considered to be a predictor of insulin initiation in patients with GDM [22]. 

The outcomes revealed that few patients in the metformin and insulin groups developed gestational HTN and pre-eclampsia. Furthermore, only the difference in the rate of pregnancy-induced HTN was significant when compared to the group treated with diet. Slagjana et al. also observed significantly more cases of pre-eclampsia in those taking metformin and insulin compared to diet [17]. On the other hand, another study concluded that there is no significant difference between all management groups regarding hypertensive complications [23].

Evidence from many studies has demonstrated that treating women with GDM using glycemic-lowering therapy is associated with a slightly earlier gestational age of delivery, as compared with treatment through diet alone [13,20,21,24]. Our data analysis confirmed this evidence. In contrast, this association was not observed in the study by Slagjana et al. when comparing metformin-treated and diet-treated patients [17].

A meta-analysis of five randomized studies comprising 1270 patients with GDM was consistent with our findings, as it concluded that metformin causes similar neonatal complications to insulin [25]. Furthermore, a recent meta-analysis of 24 studies including 4934 participants revealed a reduction in neonatal complications with the use of metformin, especially for macrosomia, LGA, NICU admission, and neonatal hypoglycemia [26].

Slagjana et al. and McGrath et al. both found a significant increase in the rate of incidence of neonatal hypoglycemia in those who used metformin compared to those who were treated through diet only [17,20]. Moreover, another study reported a similar increment in neonatal hypoglycemia; however, the association was not significant [13]. These findings are in agreement with our results. However, other studies did not observe such a difference [13,23,24], with Bashir et al. even concluding that metformin reduces the risk of neonatal hypoglycemia compared to diet alone [19]. The higher rate of newborn hypoglycemia in the metformin group can be explained by the higher average prenatal FBS and PPG in this group compared with the diet group.

Surprisingly, we found that the rates of neonatal hyperbilirubinemia and jaundice were higher when using metformin compared to a specialized diet alone, which contradicts many published studies concluding that metformin therapy has no effect on neonatal jaundice [13,20,27]. However, our results coincide with the findings of D’douza et al. and Callegari et al. [21,28]. This result could be due to the earlier gestational age at delivery and increased severity of the disease in those who were treated with metformin compared to diet alone.

In contrast to the evidence of no increased risk of neonatal respiratory distress with maternal usage of metformin during pregnancy [13,20,21,23], we found a higher rate of this outcome in the metformin group compared to the group treated through diet alone. This finding could also be interpreted according to the advanced disease and earlier delivery in this group.

Notably, SGA and lower birth weight are the most highly reported side effects when metformin is used by pregnant women [29]. Interestingly, our study revealed the opposite finding, as the rate of LGA was significantly higher with metformin treatment compared to diet alone; no difference was observed in the mean birth weight. A study from Qatar shows a comparable result (27 vs. 18%; *p* = 0.008) [28]. The older age, earlier diagnosis of GDM, and higher average blood glucose before delivery in the metformin-treated patients could explain this finding.

It has been shown previously that specific maternal factors are considered to predict several neonatal outcomes inGDM. Maternal metabolic factors such as obesity, high blood pressure, high blood glucose values in the first trimester, and high HbA1c at the diagnosis of GDM have significant associations with increased outcomes. Moreover, previous macrosomia was found to have a strong role in this context. Therefore, risk stratification of the occurrence of neonatal complications based on maternal clinical parameters could guide the strategies of GDM management and the intensification of therapy [30].

After further analysis of our data through multiple regression and adjustment of several risk factors, we determined that those treated with metformin in this study did not have an increased risk of perinatal outcomes when compared to those who underwent insulin and diet treatments. However, there was a slight but significantly increased risk of a lower score for the five-minute APGAR test after metformin treatment compared to diet treatment, which is not clinically significant and is in contrast to what has been reported in the literature [13,17,21,24].

Our study is the first study in Saudi Arabia to evaluate the influence of metformin on neonatal outcomes. Furthermore, we included many different maternal risk factors that could affect the outcomes in our analysis, which serves to strengthen our study. However, the retrospective and observational nature of our study could be one of the limitations, as it could not confirm the causality and the effects of medication on the outcomes. In addition, the lack of randomization in our study may have affected the reliability of our results. Furthermore, we did not match the groups used in the study, and some missing data could affect our results. Moreover, diet compliance by participants was not assessed, which is considered an essential part of GDM management. Finally, the number of women included in the analysis was low.

## 5. Conclusions

Metformin was found to be a safe therapy for GDM, compared to insulin therapy, when considering the risk of neonatal complications. While the incidences of LGA, neonatal hypoglycemia, neonatal hyperbilirubinemia, and respiratory distress were higher with metformin therapy compared to lifestyle modification, metformin did not increase the risk of these complications after multiple regression analysis. Further prospective randomized studies in the Saudi population with long follow-up periods are required to confirm the safety of metformin with regard to the offspring of women with GDM.

## Figures and Tables

**Table 1:** Baseline characteristics for women with gestational diabetes mellitus according to treatment group.

Maternal Characteristics	Total (234)	Metformin (78)	Insulin (78)	Diet (78)	Metformin vs. Insulin *p* Value	Metformin vs. Diet Only *p* Value
Maternal age (years ± SD)	33.09 ± 5.57	33.88 ± 5.67	34.59 ± 5.36	30.79 ± 4.97	0.426	0.002 ^a^
Maternal BMI (Kg/m^2^ ± SD)	33.34 ± 5.47	34.29 ± 5.58	33.16 ± 5.64	32.55 ± 5.12	0.214	0.044 ^a^
Parity	2.67 ± 2.05	2.58 ± 1.99	3.09 ± 2.28	2.32 ± 1.8	0.137	0.24
Diagnosis of GDM (weeks ± SD)	27.16 ± 4.51	26.47 ± 4.62	26.84 ± 4.43	28.17 ± 4.36	0.612	0.02 ^a^
Previous GDM, *n* (%)	80 (34.2)	27 (34.60)	40 (51.3)	13 (16.70)	0.035 ^a^	0.01 ^a^
Family history of T2DM, *n* (%)	46 (19.7)	14 (17.90)	22 (28.20)	10 (12.80)	0.128	0.375
Previous macrosomia, *n* (%)	10 (4.3)	3 (3.80)	7 (9.00)	0	0.191	0.08
Previous abortion, *n* (%)	92 (39.3)	32 (41)	28 (35.90)	32 (41)	0.51	1
Pre-pregnancy HTN, *n* (%)	0	0	0	0	NA ^b^	NA ^b^
Pregnancy-induced HTN, *n* (%)	7 (3)	4 (5.1)	3 (3.8)	0	0.699	0.043 ^a^
Pre-eclampsia, *n* (%)	5 (2.1)	3 (3.8)	2 (2.6)	0	0.649	0.08
OGTT 0 h (mmol/L ± SD)	5.34 ± 1.19	5.27 ± 0.85	5.83 ± 1.65	4.94 ± 0.7	0.01 ^a^	0.008 ^a^
OGTT 1 h (mmol/L ± SD)	10.87 ± 1.85	10.73 ± 1.95	11.39 ± 2.07	10.54 ± 1.54	0.043 ^a^	0.489
OGTT 2 h (mmol/L ± SD)	9.16 ± 2.11	9.42 ± 2.13	9.42 ± 2.05	8.67 ± 2.07	0.991	0.027 ^a^
Gestational age at initiation of pharmacotherapy (weeks ± SD)	31.46 ± 4.55	30.88 ± 4.65	32.04 ± 4.4	NA	0.114	NA
Total daily dose of metformin reached (mg ± SD)	NA	1166.03 ± 486.61	NA	NA	NA	NA
Total daily dose of insulin reached (units ± SD)	NA	NA	16.88 ± 16.54	NA	NA	NA
Average HbA1c before delivery (%)	6.41 ± 3.7	5.7	6.44 ± 1.12	6.41 ± 4.78	0.525	0.885
Average FBS before delivery (mmol/L ± SD)	5.27 ± 1	5.42 ± 0.73	5.54 ± 1.29	4.69 ± 0.58	0.49	0.000 ^a^
Average 2 h PP before delivery (mmol/L ± SD)	7.23 ± 1.53	7.7 ± 1.38	7.53 ± 1.69	6.1 ± 0.74	0.519	0.000 ^a^
Gestational age at delivery (weeks ± SD)	38 ± 1.67	37.69 ± 2	37.6 ± 1.24	38.72 ± 1.44	0.737	0.000 ^a^

SD: standard deviation, BMI: body mass index, GDM: gestational diabetes mellitus, T2DM: type 2 diabetes mellitus, HTN: hypertension, OGTT: oral glucose tolerance test, HbA1c: glycated hemoglobin, FBS: fasting blood sugar, PP: postprandial, NA: not applicable. ^a^ *p* ≤ 0.05. ^b^ *p* value could not be calculated because of zero cell values.

**Table 2:** Neonatal outcomes for women receiving metformin in comparison to those receiving insulin or a specialized diet alone.

Parameter	Metformin	Insulin	Diet	Overall *p* Value	Metformin vs. Insulin *p* Value	Metformin vs. Diet Only *p* Value
Preterm birth (%)	10.30	14.10	5.10	0.168	0.463	0.229
NICU admission (%)	17.90	20.5	9	0.116	0.685	0.101
Neonatal hypocalcemia (%)	2.60	6.40	1.30	0.186	0.246	0.56
Neonatal hypoglycemia (%)	9	10.30	1.30	0.056	0.786	0.029 ^a^
Congenital anomaly (%)	10.30	11.50	10.30	0.956	0.797	1
Birth injury (%)	0	0	2.60	0.133	NA ^b^	0.155
Birth weight (g ± SD)	3139.27 ± 632.59	3200.58 ± 454.85	3188.26 ± 413.11	0.729	0.488	0.568
Perinatal death (%)	0	2.6	0	0.366	0.316	NA ^b^
Five-minute APGAR score ± SD	8.88 ± 0.36	8.84 ± 0.84	8.99 ± 0.3	0.257	0.698	0.055
Macrosomia (%)	2.60	2.60	0	0.362	1	0.155
Shoulder dystocia (%)	0	1.30	1.30	0.604	0.316	0.316
Neonatal jaundice/hyperbilirubinemia (%)	26.9	23.10	14.1	0.134	0.579	0.047 ^a^
Respiratory distress (%)	9	12.80	1.3	0.023 ^a^	0.441	0.029 ^a^
Large for gestational age (>90th percentile) (%)	9	12.80	1.3	0.023 ^a^	0.441	0.029 ^a^
Small for gestational age (<10th percentile) (%)	3	2.1	2.6	0.835	0.548	0.772

SD: standard deviation, NICU: neonatal intensive care unit, NA: not applicable. ^a^ *p* ≤ 0.05. ^b^ *p* value could not be calculated because of zero cell values.

**Table 3:** Multiple regression analysis of the effect of metformin on neonatal outcomes in comparison with insulin or diet alone.

Parameter	Metformin vs. Insulin *p* Value; Adjusted OR ^d^ (95%CI)	Metformin vs. Diet Only *p* Value; Adjusted OR ^e^ (95%CI)
Preterm birth	0.702; 1.25 (0.39–3.98)	0.304; 2.04 (0.53–7.69)
NICU admission	0.465; 1.42 (0.55–3.66)	0.233; 1.89 (0.66–5.56)
Neonatal hypocalcemia	0.871; 0.85 (0.12–6.21)	0.928; 0.88 (0.05–14.29)
Neonatal hypoglycemia	0.852; 1.13 (0.31–4.19)	0.120; 5.88 (0.63–50)
Congenital anomaly	0.774; 0.85 (0.27–2.61)	0.494; 1.47 (0.48–4.55)
Birth injury	NA	0.511; 0.34 (0.01–8.3)
Birth weight	0.941; 0.01	0.126; −0.13 ^c^
Perinatal death	0.177; 3.74 (0.55–25.39)	NA ^b^
Five-minute APGAR score	0.641; 0.04	0.036 ^a^; −0.18 ^c^
Macrosomia	0.81; 1.3 (0.15–11.1)	0.268; 2.86 (0.44–20)
Shoulder dystocia	NA	0.103; 0.08 (0.004–1.67)
Neonatal jaundice/hyperbilirubinemia	0.280; 1.55 (0.7–3.45)	0.140; 1.92 (0.81–4.55)
Respiratory distress	0.655; 0.77 (0.24–2.45)	0.102; 6.25 (0.69–50)
Large for gestational age (>90th percentile)	0.706; 0.8 (0.24–2.61)	0.152; 5.26 (0.54–50)
Small for gestational age (<10th percentile)	0.874; 1.1 (0.33–3.73)	0.575; 1.43 (0.42–4.76)

NICU: neonatal intensive care unit, NA: not applicable. ^a^ *p* ≤ 0.05. ^b^ OR could not be calculated because of zero cell values. ^c^ Adjusted standardized beta. ^d^ Adjusted for OGTT at 0, OGTT at 1 h, and previous GDM. ^e^ Adjusted for age at pregnancy, maternal BMI, OGTT at 0, OGTT at 2 h, and previous GDM.

## Data Availability

The data supporting the findings of this study are available from the corresponding author upon reasonable request.
